# Iron-based compounds coordinated with phospho-polymers as biocompatible probes for dual ^31^P/^1^H magnetic resonance imaging and spectroscopy

**DOI:** 10.1038/s41598-024-54158-x

**Published:** 2024-02-15

**Authors:** Lucie Kracíková, Ladislav Androvič, David Červený, Natalia Jirát-Ziółkowska, Michal Babič, Monika Švábová, Daniel Jirák, Richard Laga

**Affiliations:** 1 Institute of Macromolecular Chemistry, Czech Academy of Sciences, Heyrovského nám. 2, 162 00 Prague 6, Czech Republic; 2 Faculty of Chemical Technology, University of Chemistry and Technology, Prague, Technická 5, 166 28 Prague 6, Czech Republic; 3 Institute for Clinical and Experimental Medicine, Vídeňská 1958/9, 140 21 Prague 4, Czech Republic; 4 Institute of Biophysics and Informatics, First Faculty of Medicine, Charles University, Kateřinská 1660/32, 121 08 Prague, Czech Republic; 5 Faculty of Health Studies, Technical University of Liberec, Studentská 1402/2, 46117 Liberec, Czech Republic

**Keywords:** Bioconjugate chemistry, Coordination polymers, Polymer characterization, Polymer synthesis, Biophysical methods, Magnetic properties and materials, Nanoparticles

## Abstract

In this work, we present the synthesis and evaluation of magnetic resonance (MR) properties of novel phosphorus/iron-containing probes for dual ^31^P and ^1^H MR imaging and spectroscopy (MRI and MRS). The presented probes are composed of biocompatible semitelechelic and multivalent phospho-polymers based on poly(2-methacryloyloxyethyl phosphorylcholine) (pMPC) coordinated with small paramagnetic Fe^3+^ ions or superparamagnetic maghemite (*γ*-Fe_2_O_3_) nanoparticles via deferoxamine group linked to the end or along the polymer chains. All probes provided very short ^1^H *T*_1_ and *T*_2_ relaxation times even at low iron concentrations. The presence of iron had a significant impact on the shortening of ^31^P relaxation, with the effect being more pronounced for probes based on *γ*-Fe_2_O_3_ and multivalent polymer. While the water-soluble probe having one Fe^3+^ ion per polymer chain was satisfactorily visualized by both ^31^P-MRS and ^31^P-MRI, the probe with multiple Fe^3+^ ions could only be detected by ^31^P-MRS, and the probes consisting of *γ*-Fe_2_O_3_ nanoparticles could not be imaged by either technique due to their ultra-short ^31^P relaxations. In this proof-of-principle study performed on phantoms at a clinically relevant magnetic fields, we demonstrated how the different forms and concentrations of iron affect both the ^1^H MR signal of the surrounding water molecules and the ^31^P MR signal of the phospho-polymer probe. Thus, this double contrast can be exploited to simultaneously visualize body anatomy and monitor probe biodistribution.

## Introduction

Magnetic resonance imaging and spectroscopy (MRI and MRS) are advanced non-invasive techniques used in clinical and experimental medicine to visualize anatomical structures, analyze the chemical composition of tissues and metabolites, and monitor changes in physiological and pathological processes within the body. Currently, hydrogen (^1^H) is the most frequently imaged element in clinical MR methods because it is highly abundant in the human body (~ 10% of body mass), especially in water and fat, and its nucleus has favorable intrinsic physical properties spin quantum number (*s* = ½) and large gyromagnetic moment (*γ* = 42.6 MHz T^−1^) that is higher than any other isotopes, which allows its sensitive detection at relatively low magnetic fields of clinical scanners (1.5–3 T). Moreover, changes in the physicochemical properties of water correlate well with changes in biochemical processes in living tissue^1^.

To increase the contrast and improve the visibility of specific internal body areas, a contrast agent may be administrated to the patient before the examination^2^. The most commonly used type of contrast agents in clinical ^1^H-MR settings are paramagnetic coordination compounds based on gadolinium, which enhance the MR signal by dominant shortening the *T*_1_ relaxation time of nearby water protons^3^. Although these agents significantly improve the diagnostic capabilities of MRI, their use has been associated with some adverse effects such as allergic reactions, nephrotoxicity and rarely nephrogenic systemic fibrosis^4^. For some specific purposes, such as imaging the liver, transplanted cells or a certain type of cancer, superparamagnetic iron oxide-based nanoparticles, also called SPIONs (usually based on magnetite—Fe_3_O_4_), are clinically used as safer alternatives^5–8^. However, despite their biogenic nature, there are concerns that these *T*_1_/*T*_2_ relaxants also exhibit toxic effects *in vivo*, primarily due to non-specific binding to cellular components and blood plasma proteins, unless their surface is effectively coated, e.g. with hydrophilic polymers^9^. High hopes are also placed on low molecular weight iron(III) complexes, which have the advantage of high thermodynamic stability, low long-term toxicity and potential for multimodal imaging^10^. On the other hand, iron is a redox-active metal that can induce the production of dangerous reactive oxygen species in the body, so it is necessary to ensure its bioinertness by using a suitable ligand or a protective polymer layer^11,12^. Therefore, there is a growing need to The development of bioinert and tissue-specific contrast agents for a safer and more efficient contrast agent for in vivo MR imaging is therefore an important challenge.

While ^1^H-MR provides a unique insight into the anatomical distribution and morphology of organs and tissues within the body, advanced MR techniques have recently been developed to visualize the nuclei of other elements (called X-nuclei) to gather valuable information about tissue function, metabolism and physiology^13^. Among various imageable X-nuclei, such as ^11^B, ^19^F, ^23^Na, ^31^P, etc., ^31^P has received a considerable attention in recent years because it is present in many biomolecules such as nucleic acids, phospholipids, bioenergetic molecules, etc., which play an important role in many vital processes. Although the concentration of phosphorus in the human body is ~ 10 times lower (~ 1% of body mass) than that of hydrogen, and its monoisotope ^31^P has ~ 2.5 times lower gyromagnetic moment (*s* = ½, *γ* = 17.2 MHz T^−1^), and therefore lower MR sensitivity than the proton ^1^H, clinical scanners equipped with sensitive radiofrequency coils together with advanced imaging sequences (e.g. chemical shift imaging) allow its reliable detection. In clinical practice, ^31^P MR techniques are mainly used to study cell membrane composition, phosphorylated metabolite levels, bioenergetic status of organs and tissues, and intracellular pH levels^14–16^. Moreover, ^31^P MR techniques are also able to follow the biological fate of various exogenous phosphorus-containing compounds such as drug delivery carriers or biosensors of pathological conditions, which, in synergy with ^1^H MR, can provide comprehensive information on their biodistribution and pharmacokinetics or on the occurrence of physiological abnormalities in the body^17,18^.

In order to effectively integrate ^1^H with ^31^P MRI and thus take full advantage of the information provided by both methods, it is desirable to use a contrast agent/probe that would improve the visibility of internal body structures in ^1^H MRI while ensuring its traceability by ^31^P MRI. Such a compound should optimally contain in its structure both a (super)paramagnetic metal amplifying the ^1^H MR signal of nearby water protons and phosphorus in its structure to amplify the ^1^H MR signal of nearby water protons as well as to providing high and distinguishable ^31^P MR signal. In addition, the presence of paramagnetic metal can also affect the relaxation of ^31^P nuclei, allowing a ^31^P MR signal of sufficient intensity to be obtained^19,20^. In terms of clinical application, the contrast agent/probe while it should be well tolerated by the organism, have a It is also advantageous if the contrast agent/probe has a long biological half-life, specifically accumulate in target tissues, and should be metabolized or eliminated from the body after performing its function. One material that could meet these requirements is hydrophilic biocompatible phospho-polymers or phosphate-based colloids coordinated with iron.

In this regard, the most frequently studied phospho-polymers are water-soluble poly(organophosphazene)s, poly(phosphoester)s, or poly(2-methacryloyloxyethyl phosphorylcholine) (pMPC), all experimentally applied in many biomedical fields, including drug delivery, regenerative medicine, antifouling coating technology or biosensing^21–23^. However, since The results of our recent comparative study of chemically and structurally different phospho-polymers showed that the best results in terms of MR properties are achieved by linear pMPC achieved the best results in terms of MR properties, we decided to focus just on this type of polymer^17^. We believe that the favorable MR properties of the pMPC polymer are due to both the high content of phosphorus in its structure and the polyzwitterionic character ensuring the very high hydrophilicity of its chains. In addition, the linear arrangement of its chains allows the anchoring of chelating groups both at the end and along the backbone, which can effectively control the concentration of coordinated Fe^3+^ ions or the coverage density of SPION nanoparticles and thereby influence their colloidal and biological stability. Other advantages of Finally, pMPC include that it can be prepared relatively easily and reproducibly by controlled polymerization techniques which not only that provide a well-defined material with tunable structural properties^24^. But also allow the incorporation of ligands for iron complexation.Therefore, we have focused on this type of polymer in this work.

For these purposes, two types of pMPC-based polymers were synthesized in this work: (i) semitelechelic with one iron-chelating deferoxamine (DFA) group at the end of the polymer chain and (ii) multivalent with multiple DFA groups distributed along the polymer chain. These polymers were complexed with small paramagnetic Fe^3+^ ions or colloidal superparamagnetic maghemite (*γ*-Fe_2_O_3_) nanoparticles. The influence of the density (single vs multiple) and form (soluble vs colloidal) of iron complexed to MPC-based polymers on ^31^P and ^1^H MR properties was evaluated under experimental conditions on a 4.7 T MR scanner with the aim of finding the optimal composition of complexes trackable in vitro/in vivo using dual ^1^H/^31^P MR spectroscopy and imaging. We believe that the development of new biosensors based on complexes of phospho-polymers with iron has great potential to expand the application possibilities of MR techniques usable in both anatomical and functional imaging.

## Materials and methods

### Chemicals

3-Aminopropanoic acid, 4,4′-azobis(4-cyanopentanoic acid) (ACVA), 2,2′-azobis(2-methylpropionitrile) (AIBN), *N,N'*-dicyclohexylcarbodiimide (DCC), 4-dimethylaminopyridine (DMAP), methacryloyl chloride, 2-methacryloyloxyethyl phosphorylcholine (MPC), thiazolidine-2-thione (TT) and triethylamine (TEA) were purchased from TCI Europe, Belgium. Ammonium hydroxide, 4-cyano-4-(phenylcarbonothioylthio)pentanoic acid (CPP), 2-cyano-2-propyl benzodithioate (CPB), deferoxamine methanesulphonate salt (DFA), iron(III) chloride hexahydrate, iron(II) chloride tetrahydrate and sodium bicarbonate were purchased from Sigma-Aldrich, Czech Republic. 2,2'-Azobis(4-methoxy-2,4-dimethylvaleronitrile) (V-70) was from FUJIFILM Wako Chemicals Europe, Germany. All solvents were of HPLC grade (obtained from VWR International, Czech Republic) and dried over a layer of activated molecular sieves (4 Å) before use.

### Synthesis of maghemite nanoparticles

Maghemite nanoparticles (*γ*-Fe_2_O_3_) were produced by aqueous co-precipitation of FeCl_3_∙6H_2_O and FeCl_2_∙4H_2_O salts in the presence of ammonium hydroxide under sonication followed by oxidation of the generated magnetite (Fe_3_O_4_) with sodium hypochlorite^9^. The z-average hydrodynamic diameter determined by Dynamic Light Scattering (DLS) (*D*_*h*_^DLS^) and the number-average diameter determined by Transmission Electron Microscopy (TEM) (*D*_*n*_^TEM^) of *γ*-Fe_2_O_3_ nanoparticles were 60.9 nm and 10.3 nm, respectively.

### Synthesis of monomer

*3-(3-Methacrylamidopropanoyl)thiazolidine-2-thione (Ma-βAla-TT)* monomer was synthesized by the acylation of 3-aminopropanoic acid with methacryloyl chloride in an aqueous alkaline medium followed by the reaction of formed 3-methacrylamidopropanoic acid with TT in tetrahydrofuran in the presence of DCC and DMAP^25^.

### Synthesis of RAFT agent and initiator

*2-cyano-5-oxo-5-(2-sulphanylidene-1,3-thiazolidin-3-yl)pentan-2-yl benzenecarbodithioate (CTA-TT)* RAFT agent was synthesized by the condensation of CPP acid with TT in dichloromethane in the presence of DCC and DMAP^26^.

*2-[1-Cyano-1-methyl-4-oxo-4-(2-thioxo-thiazolidin-3-yl)-butylazo]-2-methyl-5-oxo-5-(2-thioxothiazolidin-3-yl)-pentanenitrile (ACVA-(TT)*_*2*_*)* initiator was prepared by the reaction of ACVA with TT in tetrahydrofuran in the presence of DCC and DMAP^26^.

### Synthesis of polymers

(**1a**) *Semitelechelic CN-p(MPC)-DFA* polymer was prepared in three synthetic steps as follows: First, a mixture of CTA-TT (15.8 mg, 41.7 µmol) and ACVA-(TT)_2_ (10 mg, 20,8 µmol) was dissolved in DMSO (0.4 mL) and added to a solution of MPC (500.0 mg, 1.7 mmol) in methanol (1.5 mL). The reaction mixture was thoroughly bubbled with argon and polymerized in a sealed glass ampoule at 70 °C for 16 h. After cooling to room temperature, the polymer was obtained by precipitation of the reaction mixture into acetone (40 mL) and purified by subsequent re-precipitation from methanol into acetone (40 mL). Centrifugation and vacuum drying of the precipitate yielded 400 mg (77%) of DTB-p(MPC)-TT polymer precursor as a pink powder. The number-average molecular weight (*M*_n_) and the dispersity (*Ð*^SEC^) of the polymer were 18.1 kg mol^−1^ and 1.01, respectively.

Next, a mixture of DTB-p(MPC)-TT (150.0 mg, 7.6 µmol DTB groups) and AIBN (25.0 mg, 152 µmol) was dissolved in methanol (1.5 mL) and allowed to react at 80 °C for 2 h. The reaction mixture was cooled down to room temperature and the polymer was obtained by precipitation into acetone (40 mL). After purification on a column filled with Sephadex LH-20 in methanol, the polymer was isolated by precipitation into acetone and dried in vacuo to give 100 mg (67%) of CN-p(MPC)-TT polymer precursor as a yellow amorphous powder. The *M*_n_ and *Ð* of the polymer were 19.2 kg mol^−1^ and 1.02, respectively. The content of TT end groups of the polymer was 51.0 µmol g^−1^, corresponding to ~ 0.98 TT groups per polymer chain.

Finally, a mixture of DFA (14.2 mg, 25.3 µmol) and TEA (7.0 µL, 50.3 µmol) was dissolved in DMSO (0.330 mL) under heat, added to a solution of CN-p(MPC)-TT (100 mg, 5.1 µmol TT) in methanol (0.660 mL), and the mixture was allowed to react for 48 h at room temperature. After purification on a column filled with Sephadex LH-20 in methanol, the polymer was isolated by precipitation into acetone and dried in vacuo to give 49 mg (49%) of the resulting CN-p(MPC)-DFA polymer as a white powder. The *M*_n_ and *Ð*^SEC^ of the polymer were 19.7 kg mol^−1^ and 1.02, respectively. The content of DFA end groups of the polymer was 49.7 µmol g^−1^, corresponding to ~ 0.98 DFA groups per polymer chain.

(**1b**) *Multivalent CN-p(MPC-co-Ma-βAla-DFA)-CN* polymer was prepared in three synthetic steps as follows: First, a mixture of CPB (8.0 mg, 36.6 µmol) and V-70 (5.6 mg, 18.3 µmol) was dissolved in DMSO (0.3 mL) and added to a solution of MPC (400 mg, 1.4 mmol) and Ma-βAla-TT (39.0 mg, 0.2 mmol) in methanol (1.2 mL). The reaction mixture was thoroughly bubbled with argon and polymerized in a sealed glass ampoule at 40 °C for 48 h. After cooling to room temperature, the polymer was obtained by precipitation of the reaction mixture into acetone–diethyl ether (1:1, 40 mL) and purified by subsequent re-precipitation from methanol into the same precipitant. Centrifugation and vacuum drying of the precipitate yielded 350 mg (78%) of DTB-p(MPC-*co*-Ma-βAla-TT)-CN polymer precursor as an orange powder. The *M*_n_ and the *Ð*^SEC^ of the polymer were 12.7 kg mol^−1^ and 1.10, respectively.

Next, a mixture of DTB-p(MPC-*co*-Ma-βAla-TT)-CN (350.0 mg, 26.9 µmol DTB groups) and AIBN (88.2 mg, 537.2 µmol) was dissolved in methanol (3.5 mL) and allowed to react at 80 °C for 2 h. The reaction mixture was cooled down to room temperature and the polymer was obtained by precipitation into acetone–diethyl ether (1:1, 40 mL). After purification on a column filled with Sephadex LH-20 in methanol, the polymer was isolated by precipitation into acetone–diethyl ether (1:1) and dried in vacuo to give 262 mg (75%) of CN-p(MPC-*co*-Ma-βAla-TT)-CN polymer precursor as a yellow amorphous powder. The *M*_n_ and *Ð*^SEC^ of the polymer were 13.0 kg mol^−1^ and 1.10, respectively. The content of TT groups along the polymer chain was 7.7 mol%.

Finally, a mixture of DFA (45.2 mg, 69.8 μmol) and TEA (9.5 µL, 69.8 μmol) was dissolved in dimethylacetamide (3.4 mL) under heat, added to a solution of CN-p(MPC-*co*-Ma-βAla-TT)-CN (262.0 mg, 69.8 mmol TT) in methanol (6.8 mL), and the mixture was allowed to react for 48 h at room temperature. After purification on a column filled with Sephadex LH-20 in methanol, the polymer was isolated by precipitation into acetone–diethyl ether (1:1, 40 mL) and dried in vacuo to give 165 mg (63%) of the resulting CN-p(MPC-*co*-Ma-βAla-DFA)-CN polymer as a white powder. The *M*_n_ and *Ð*^SEC^ of the polymer were 12.3 kg mol^−1^ and 1.10, respectively. The content of DFA groups along the polymer chain was 4.9 mol%.

### Complexation of polymers with Fe^3+^ ions

FeCl_3_∙6H_2_O (5 eq.) was added to a solution of DFA groups-containing polymer **1a** or **1b** (1 eq.) in methanol (10% w/v) and allowed to react for 1 h at room temperature. After purification on a column filled with Sephadex LH-20 in methanol, the polymer/Fe^3+^ complexes were isolated by precipitation into acetone–diethyl ether (1:1) and dried in vacuo to form a brownish red powder product. The *M*_n_ and *Ð*^SEC^ were 20.8 kg mol^−1^ and 1.10 for the complexes **1a/Fe**^**3+**^, and 14.8 kg mol^−1^ and 1.03 for the complexes **1b/Fe**^**3+**^.

### Complexation of polymers with γ-Fe_2_O_3_ nanoparticles

A sterile stock solution of neat *γ*-Fe_2_O_3_ nanoparticles in water for injections was drop-wise added to an aqueous solution of DFA groups-containing polymers **1a** or **1b** (4:1 w/w) under sonication with a 1-mm^2^ tipped ultrasonic horn (Bandelin UW 3200, Germany). The final concentration of polymer/*γ*-Fe_2_O_3_ complexes was 0.5 mg mL^−1^ and the total volume of the sample was 2.0 mL. All reactions were performed in a sterile box, with the prepared polymer/*γ*-Fe_2_O_3_ complexes stored in septum-sealed vials to prevent bacterial contamination. The *D*_h_^*DLS*^ and *D*_n_^*TEM*^ were 102.3 nm and 11.8 nm for the complexes **1a/*γ*-Fe**_**2**_**O**_**3**_, and 81.8 nm and 11.3 nm for the complexes **1b/*γ*-Fe**_**2**_**O**_**3**_.

### UV–VIS spectrophotometry

Spectrophotometric analysis of functionalized polymers was carried out in quartz glass cuvettes on a Specord Plus UV–VIS spectrophotometer (Analytik Jena, Germany). The molar content of DTB and TT groups in the polymers was determined at 302 and 305 nm in methanol using the molar absorption coefficient of 12 100 and 10 300 L mol^−1^ cm^−1^, respectively^26^. DFA group content was measured indirectly at 432 nm upon addition of FeCl_3_∙6H_2_O (2 eq. compared to the theoretical content of DFA groups) to the aqueous solution of polymers using a molar absorption coefficient of 2 585 L mol^−1^ cm^−1^^27^. The resulting values of functional group contents were obtained by arithmetic average of 3 independent measurements.

### Size-exclusion chromatography

The number- and weight-averages of molecular weights (*M*_n_ and *M*_w_) and dispersities (*Ð*^SEC^, *Ð*^SEC^ = *M*_w_*/M*_n_) of the polymers were determined by size-exclusion chromatography (SEC) on a HPLC system (Shimadzu, Japan) equipped with an internal UV–VIS diode array detector (SPD-M20A), an external differential refractometer (Optilab T-rEX), and a multi-angle light-scattering detector (DAWN HELEOS II, both Wyatt Technology, USA). TSKgel SuperAW3000 and SuperAW4000 columns (Tosoh Bioscience, USA) in series were used to analyze samples in the mobile phase of 80% methanol and 20% sodium acetate buffer (0.3 M, pH 6.5) at a flow rate of 0.6 mL·min^−1^. The d*n*/d*c* values of 0.125 mL g^−1^ were used to calculate the molecular weights of the MPC-based polymers^18^. The resulting molecular weight values were obtained by arithmetic average of 2 independent analyses.

### Dynamic light scattering

The z-averages of the hydrodynamic diameters (*D*_h_^DLS^) and the polydispersity indexes (PDI) of neat *γ*-Fe_2_O_3_ nanoparticles and polymer/*γ*-Fe_2_O_3_ complexes were determined by the DLS technique at a scattering angle of 173° using a Nano-ZS instrument (Malvern Instruments, UK) equipped with a 4 mW, 633 nm laser. Samples of polymer/*γ*-Fe_2_O_3_ complexes with a weight concentration of 0.5 mg mL^−1^ were measured in water and in PBS buffer (0.15 M, pH 7.4) after tenfold dilution at 25 °C. For the evaluation of the DLS data, the DTS (Nano) program was used. The resulting *D*_h_^DLS^ values were arithmetic means of at least 10 independent measurements.

### Transmission electron microscopy

The microphotographs of neat *γ*-Fe_2_O_3_ nanoparticles and polymer/*γ*-Fe_2_O_3_ complexes were measured using a transmission electron microscope (FEI-TEM, Tecnai, G2 Spirit, Oregon, USA). Ultrapure-water-diluted dispersions were directly dropped on a copper grid with a carbon film and dried at room temperature. Morphological parameters, such as the number- and weight-average of particle diameters (*D*_n_^TEM^ and *D*_w_^TEM^) and dispersity (*Ð*^TEM^, *Ð*^TEM^ = *D*_w_^TEM^/*D*_n_^TEM^), were calculated from the obtained TEM microphotographs using the following equations: ${\text{D}}_{\text{n}}\text{=}\boldsymbol{ }\sum {\text{n}}_{\text{i}}{{\text{D}}}_{\text{i}}\text{/}\sum {\text{n}}_{\text{i}}$, ${\text{D}}_{\text{w}}= \text{ } \sum {\text{n}}_{\text{i}}{{\text{D}}}_{\text{i}}^{4}\text{/}\sum {\text{n}}_{\text{i}}{{\text{D}}}_{\text{i}}^{3}$, where *n*_i_ is the number of particles and *D*_i_ is the particle diameter. Particle size analysis was performed using ImageJ software, measuring at least 300 objects in each sample. Particle diameters were measured in manual mode from multiple microphotographs of different regions in the sampling grid.

### Magnetic resonance spectroscopy and imaging

MR characterization of the polymer/iron complexes was performed in aqueous solutions using a 4.7 T scanner (Bruker BioSpin, Ettlingen, Germany) equipped with a homemade dual ^1^H/^31^P radiofrequency (RF) surface coil as previously described^18^. The measurements of the samples were performed at a normalized molar concentration of phosphorus (*c*_n_^P^) of 100 mmol L^−1^ corresponding to a weight concentration (*c*_w_^pol^) of 29.5 mg mL^−1^ for polymer **1a** and 32.0 mg mL^−1^ for polymer **1b**. The molar concentrations of iron (*c*_n_^Fe^) in the polymer/iron complexes were 0.03 mmol L^−1^ for the 1a/Fe^3+^ complex, 0.084 mmol L^−1^ for the 1b/Fe^3+^ complex and 6.26 mmol L^−1^ for both neat *γ*-Fe_2_O_3_ nanoparticles and **1a/*γ*-Fe**_**2**_**O**_**3**_ and **1b/*γ*-Fe**_**2**_**O**_**3**_ complexes. ^1^H MR images were obtained using standard 2D rapid acquisition and a relaxation enhancement (RARE) multi-spin echo MR sequence based on the following parameters: spatial resolution = 312 × 312 µm^2^, slice thickness = 2.5 mm, scan time (ST) = 1 min 16 s, *T*_2w_: repetition time (TR) = 3300 ms, echo time (TE) = 36 ms, turbo factor = 4 and *T*_1w_: TR = 294.8 ms, TE = 16.7 ms, turbo factor = 2. ^31^P MR images were obtained using a chemical shift imaging (CSI) sequence (TR = 500 ms, ST = 15 min, field of view FOV = 36 mm; resolution 2.25 × 2.25 × 5.8 mm^3^). ^31^P MRI SNR was calculated using SNR = 0.655 · *S* · *σ*^−1^, where *S* is signal intensity in the region of interest (ROI), *σ* is the standard deviation of background noise, and constant 0.655 reflects the Rician distribution of background noise in a magnitude MR image.

### Relaxometry

Relaxometric experiments of aqueous solutions of polymer/iron complexes were performed on a 1.5 T Minispec 60 MHz relaxometer (Bruker Biospin, Germany) at 37 °C. The measurements of the samples were performed at a normalized molar concentration of phosphorus (*c*_n_^P^) of 100 mmol L^−1^ corresponding to a weight concentration (*c*_w_^pol^) of 29.5 mg mL^−1^ for polymer **1a** and 32.0 mg mL^−1^ for polymer **1b**. The molar concentrations of iron (*c*_n_^Fe^) in the polymer/iron complexes were 0.03 mmol L^−1^ for the **1a/Fe**^**3+**^ complex, 0.084 mmol L^−1^ for the **1b/Fe**^**3+**^ complex and 6.26 mmol L^−1^ for both neat *γ*-Fe_2_O_3_ nanoparticles and **1a/*γ*-Fe**_**2**_**O**_**3**_ and **1b/*γ*-Fe**_**2**_**O**_**3**_ complexes. ^1^H *T*_1_ relaxation time was measured using an inversion recovery sequence (TR = 0.01–10 000 ms, recycle delay = 2 s, number of scans = 4, TE = 0.05 ms, points for fitting = 20). ^1^H *T*_2_ relaxation time was measured using the Carr-Purcell-Meiboom-Gill sequence (TR = 10 000 ms, recycle delay = 2 s, number of scans = 8, TE = 0.05 ms, points for fitting = 30 000). Relaxivities *r*_1_ and *r*_2_ were calculated using the least-squares curve fitting of *R*_1_ = 1/*T*_1_ and *R*_2_ = 1/*T*_2_ relaxation rates [s^−1^] versus iron concentration [mmol L^−1^]. ^31^P *T*_1_ relaxation time was measured using 10 spectroscopic single-pulse sequences with varying repetition times (TR = 200–4000 ms) and the ^31^P *T*_2_ relaxation time was measured using 10 spectroscopic Carr-Purcell-Meiboom-Gill (CPMG) sequences with varying echo times (TR = 5000 ms, TE = 20–1600 ms). Data were quantified by plotting amplitudes and fitting the appropriate curve (*S* ≈ *S*_0_ · (1—e^−*t*/*T*1^) for *T*_1_; *S* ≈ *S*_0_ · e^−*t*/*T*2^ for *T*_2_), where *S* is signal intensity (*S*_0_ signal intensity at equilibrium) and *t* is time: TR for *T*_1_ and TE for *T*_2_, respectively.

## Results and discussion

### Synthesis of phospho-polymer/Fe^3+^ ions complexes

Among many different types of hydrophilic phosphorus-containing polymers, we decided to use a polymer based on 2-methacryloyloxyethyl phosphorylcholine (MPC). This poly-zwitterion is characterized not only by high solubility in aqueous solutions, resistance to non-specific protein adsorption and cell adhesion, and ability to effectively penetrate cell membranes, but also by excellent ^31^P-MR properties, making it an optimal material for the purposes of this study^17,24^. In addition, MPC can be copolymerized by controlled polymerization techniques with functionalized monomers in the presence of functionalized chain transfer agents (CTA) allowing incorporation of reactive groups along or at the ends of the pMPC chains for subsequent post-polymerization modification with metal-chelating ligands^23^.

**Figure 1:**
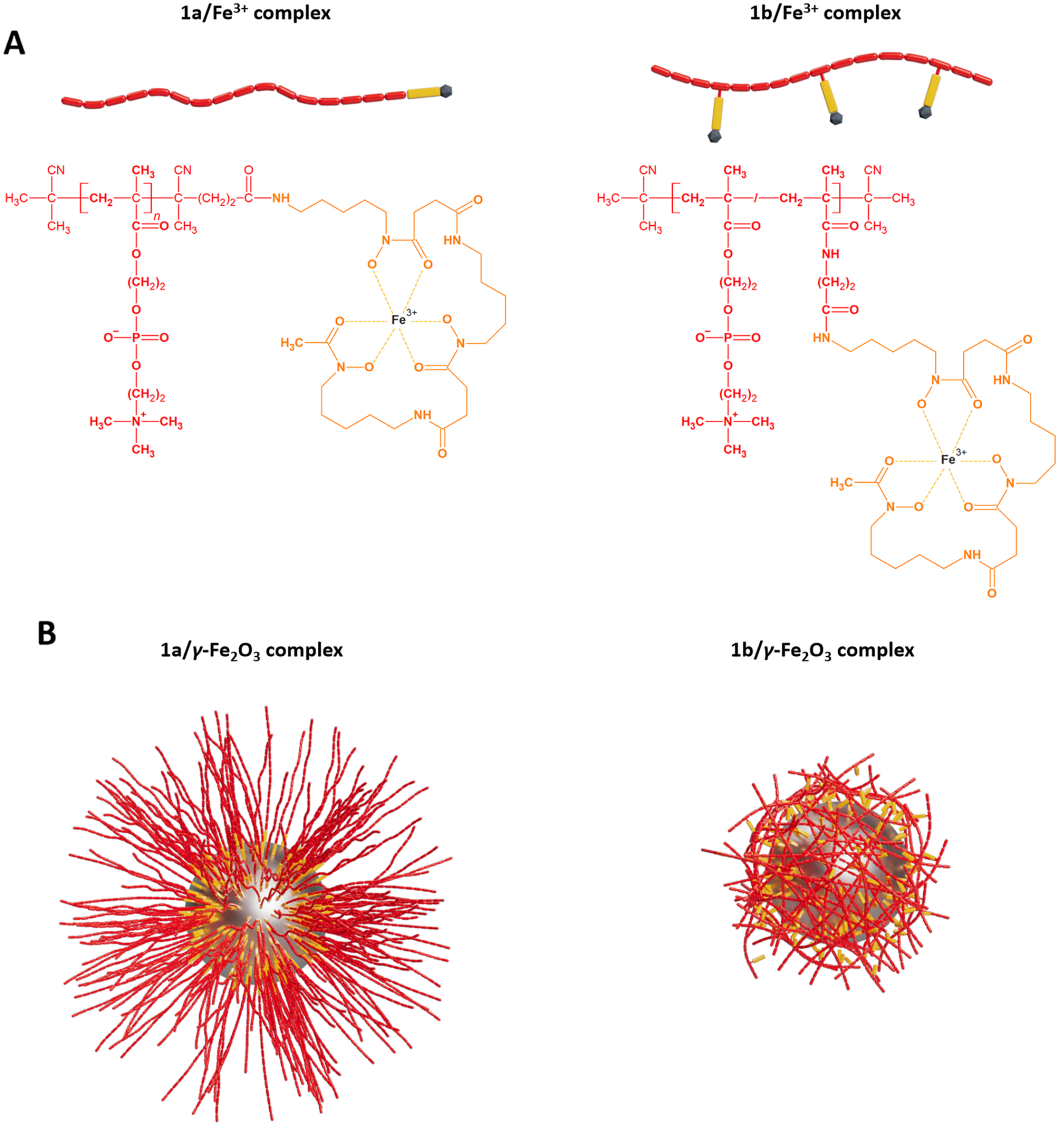
Schematic representation and chemical structures of complexes of semitelechelic polymer **1a** (left) and multivalent polymer **1b** (right) with (**A**) Fe^3+^ ions and with (**B**) *γ*-Fe_2_O_3_ nanoparticles.

In this study, we used reversible addition−fragmentation chain-transfer (RAFT) polymerization of MPC in the presence of thiazolidine-2-thione (TT) group-functionalized CTA to produce a semitelechelic homopolymer with one terminal amino-reactive TT group; and RAFT copolymerization of MPC with a TT group-containing monomer (Ma-βAla-TT) to obtain a multivalent copolymer with multiple TT groups statistically distributed along the polymer backbone. The polymers were characterized by low dispersity (*Ð* ≤ 1.1) and molecular weight in the range of ~ 12–20 kg mol^−1^, which ensures both increased accumulation in tissues with more porous vasculature (e.g. solid tumors) via the enhanced permeability and retention effect and excretion from the body via the reticuloendothelial pathway after fulfilling their function^28^. The coordination of the polymers with iron was mediated by a compound called deferoxamine (DFA). DFA is a small natural siderophore secreted by *Streptomyces pilosus*, whose primary function is to transport complex Fe^3+^ ions from the extracellular region across cell membranes^29^. Due to its strong affinity for iron (the formation constant (logβ) of the dominant species of the Fe^3+^/DFA complex in 0.1 mol L^−1^ KCl solution at 25 °C is 41.4^30^), DFA is clinically used to treat acute iron poisoning or haemochromatosis by binding free iron in the bloodstream and increasing its urinary excretion^31^. DFA was attached to the polymers by reacting its terminal amino group with the TT groups of the polymers to form an amide bond. As a source of iron, we used low-molecular-weight paramagnetic Fe^3+^ ions, which are considered one of the less toxic alternatives to clinically used Gd^3+^ ions^12^. The resulting complexes of MPC polymers with Fe^3+^ ions were obtained by incubating the DFA group-containing polymers in an aqueous FeCl_3_ solution. Neither the attachment of DFA groups nor the complexation with Fe^3+^ ions affected the solubility and molecular weights distribution of the polymers. The characteristics of the polymer/Fe^3+^ complexes are summarized in Table 1; their chemical structures are depicted in Figure 1.

**Table 1:** Size-exclusion chromatography (SEC)/UV–VIS Structural characteristics (number-average molecular weights—*M*_n_, dispersities—*Ð* and molar contents of DFA groups—*n*) of MPC-based polymers and their complexes with Fe^3+^ ions.

Sample code	Polymer structure	*M*_n_ [kg mol^−1^]	*Ð*^SEC^	*n*^DFA^ [µmol g^−1^]
1a	CN-p(MPC)-DFA	19.7	1.02	49.7
1b	CN-p(MPC-*co*-Ma-βAla-DFA)-CN	12.3	1.10	155.5
1a/Fe^3+^	CN-p(MPC)-DFA/Fe^3+^	20.8	1.03	49.7
1b/Fe^3+^	CN-p(MPC-*co*-Ma-βAla-DFA/Fe^3+^)-CN	14.8	1.10	155.5

### Synthesis of phospho-polymer/γ-Fe_2_O_3_ nanoparticle complexes

Most clinically approved iron-based contrast agents for MRI, including Feridex®, Resovist®, Lumirem®, etc., consist of various forms of superparamagnetic iron oxide nanoparticles that are characterized by high relaxivity, long-term circulation, or preferential passive accumulation in tissues with leaky vasculature, such as tumors^32^. However, the preparation of colloidally stable nanoparticles with surface properties allowing their further modification (e.g. by a phospho-polymer) is challenging. This is because nanoparticles based on bare iron oxides tend to agglomerate, and nanoparticles prepared in the presence of surfactants (e.g. oleic acid, oleylamine, dioctylamine, etc.) or hydrophilic polymers (e.g. dextran, polyethylene glycol, polylysine, etc.) to increase their stability in aqueous solutions have a protective layer around their surfaces that limits their subsequent functionalization^9,33,34^.

**Figure 2:**
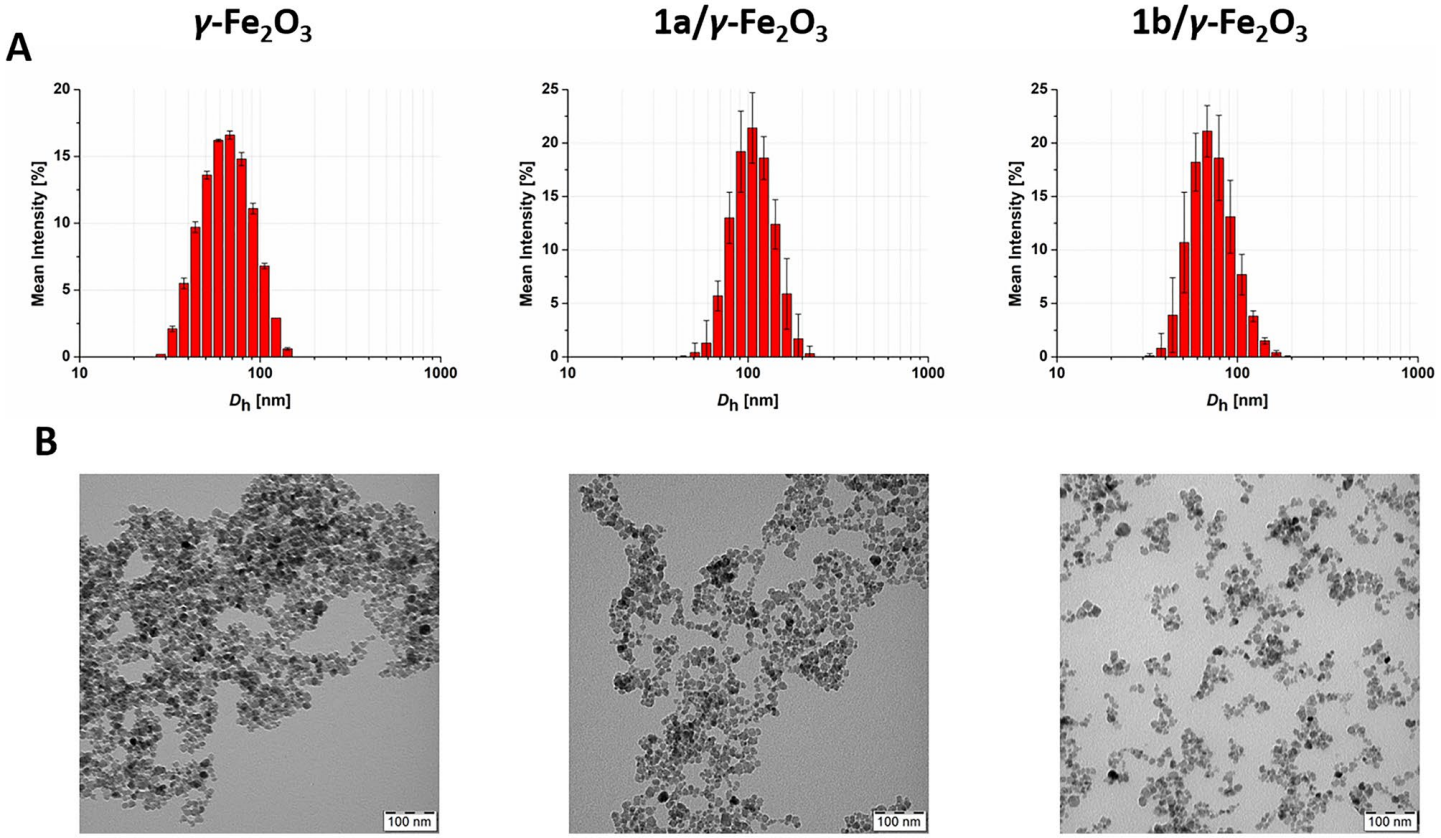
Size characterization of *γ*-Fe_2_O_3_ nanoparticles complexed with semitelechelic polymer **1a** and multivalent polymer **1b**: (**A**) DLS analysis of aqueous particle solution plotted as hydrodynamic diameter (*D*_h_) distribution by mean intensity; (**B**) TEM micrographs of dried nanoparticles.

To avoid these difficulties, we used a special co-precipitation technique of Fe^3+^ and Fe^2+^ salts to produce maghemite (*γ*-Fe_2_O_3_) nanoparticles dielectrically stabilized with sodium citrate, which are characterized not only by reproducible preparation, high colloidal stability and easy access of functionalized (macro)molecules to their surface, but also by lower activity towards reactive oxygen species (ROS) and higher saturation magnetization values compared to commonly used magnetite (Fe_3_O_4_) nanoparticles^35^. DLS and TEM analyzes showed that the prepared nanoparticles were well-defined spheroids with *D*_h_^DLS^ = 60.9 nm (PDI = 0.106) and *D*_n_^TEM^ = 10.3 nm (*Ð*^TEM^ = 1.113) (see Figure 2). In addition, elemental analysis (data not shown) proved that the *γ*-Fe_2_O_3_ nanoparticles contained a negligible amount of the citrate incorporated in their structure, which is an important precondition for subsequent surface modification.

As in the case of polymer/Fe^3+^ ion complexes, the attachment of MPC-based polymers to the *γ*-Fe_2_O_3_ nanoparticle core was mediated through DFA moieties present either at the end (**1a**) or along (**1b**) the polymer chains. Based on the results presented in our previous study^27^, a *γ*-Fe_2_O_3_ to polymer ratio of 4:1 (w/w) was used to ensure efficient coating of the nanoparticle surface, with no free polymer detected in the solution, as documented by TEM (see Figure 2). More effective coating in terms of nanoparticles size occurred when the semitelechelic polymer **1a** was used, where an increase in *D*_h_^DLS^ of the nanoparticles by more than 40 nm was observed, which was approximately twice as much as in the case of the multivalent polymer **1b**. Importantly, the hydrodynamic diameters of the nanoparticles are in range of previously clinically approved contrast agents based on superparamagnetic iron oxide (e.g. 62 nm and 150 nm)^36^. Also, the scattered light intensity (*I*_LS_^DLS^), proportional to the number (molecular weight) of bound polymer chains, increased by ~72% after coating the nanoparticles with polymer **1a**, while it only increased by ~12% in the case of polymer **1b**, indicating that polymer **1a** covered the surface of the nanoparticles more densely (see Table 2 and Figure 2). We assume that this is due to the conformation of the polymer chains in solution; while in the case of a semitelechelic polymer its chains stick out from the surface of the nanoparticles to which they are densely attached via a single point, the chains of a copolymer with multiple binding sites are tightly attached to the nanoparticle surface via cooperative interactions. A schematic representation of the polymer/*γ*-Fe_2_O_3_ nanoparticle complexes is shown in Figure 1.

**Table 2:** Dynamic light scattering (DLS) characteristics Size parameters (hydrodynamic diameters—*D*_h_, polydispersity indexes—PDI and light scattering intensities—*I*_LS_) and transmission electron microscopy (TEM) characteristics (number-average diameters—*D*_n_ and dispersities—*Ð*) of freshly prepared and 3-years old *γ*-Fe_2_O_3_ nanoparticles and polymer/*γ*-Fe_2_O_3_ complexes in water and in PBS buffer (after tenfold dilution), respectively. The standard deviations of the variables obtained from the DLS measurements were ≤ 3%.

	Sample Code	*D*_h_^DLS^ [nm]	PDI^DLS^	*I*_LS_^DLS^ [k counts s^−1^]	*D*_n_^TEM^ [nm]	*Ð*^TEM^
Fresh, in H_2_O	*γ*-Fe_2_O_3_	60.9	0.106	92.5	10.3	1.113
	1a/*γ*-Fe_2_O_3_	102.3	0.077	159.0	11.8	1.142
	1b/*γ*-Fe_2_O_3_	81.8	0.101	103.5	11.3	1.145
3 yrs old, in H_2_O	*γ*-Fe_2_O_3_	58.1	0.122	101.1	n.d.^i^	n.d
	1a/*γ*-Fe_2_O_3_	100.5	0.107	64.8	n.d	n.d
	1b/*γ*-Fe_2_O_3_	100.6	0.120	93.4	n.d	n.d
3 yrs old, diluted with PBS	*γ*-Fe_2_O_3_	n.d	n.d	n.d	n.d	n.d
	1a/*γ*-Fe_2_O_3_	93.3	0.101	38.0	n.d	n.d
	1b/*γ*-Fe_2_O_3_	95.3	0.114	27.8	n.d	n.d

^i^Not detectable.

In order to most reliably describe the changes in size and morphology of *γ*-Fe_2_O_3_ nanoparticles coordinated to polymers without the influence of time and environmental, we monitored their properties immediately after complexation in pure water. However, for the purposes of the considered application, it was also interesting to see whether the nanoparticles would exhibit the same characteristics over time and under conditions simulating the physiological environment. Therefore, we performed DLS analysis of neat *γ*-Fe_2_O_3_ nanoparticles and polymer/*γ*-Fe_2_O_3_ nanoparticle complexes approximately 3 years after their preparation in both pure water and in PBS (Table 1). All types of nanoparticles prepared in pure water showed excellent colloidal stability even after three years of storage, documented by small changes in hydrodynamic sizes and low polydispersity indexes. In the case of the complex of semitelechelic polymer with *γ*-Fe_2_O_3_ nanoparticles (**1a/*γ*-Fe**_**2**_**O**_**3**_), however, there was an almost 2.5-fold decrease in the intensity of scattered light (*I*_LS_^DLS^) compared to the freshly prepared complex, which could be explain by the fact that a part of the polymer chains, linked to the nanoparticles through a single point, was detached from the surface and released into the solution over time. This theory is supported by only about a 10% decrease in *I*_LS_^DLS^ for complexes with multivalent polymer (**1b/*γ*-Fe**_**2**_**O**_**3**_), which are attached to the nanoparticle surface via multiple points through a cooperative interaction and their cleavage during storage is therefore less likely. This corresponds to an increase in the size of these complexes over time, where the bonding between the DFA groups of the polymer and the particle surface is probably (at least) partially broken, causing a conformational change of the polymer chains, which then protrude from the particle surface similarly to the chains of the semitelechelic polymer. Tenfold dilution of aqueous solutions of nanoparticles with phosphate buffer (0.15 M PBS, pH 7.4) led to aggregation and subsequent sedimentation of neat nanoparticles, but their complexes with polymers still formed homogeneous dispersions with size < 100 nm and low PDI, indicating their high colloidal stability in conditions mimicking physiological environment.

### MR properties of phospho-polymer/iron complexes

When examining both types of polymer (**1a/Fe**^**3+**^and **1b/Fe**^**3+**^) and colloidal (**1a/*γ*-Fe**_**2**_**O**_**3**_ and **1b/*γ*-Fe**_**2**_**O**_**3**_) probes, a significant effect of iron manifested by a shortening of ^1^H relaxation times was observed. The resulting ^1^H relaxivity ratio (*r*_2_/*r*_1_) for probes coordinated with small paramagnetic Fe^3+^ ions (**1a/Fe**^**3+**^and **1b/Fe**^**3+**^) was in both cases approximately 1.7, indicating a slightly pronounced *T*_2_ effect, while in the case of probes coordinated with *γ*-Fe_2_O_3_ nanoparticles (**1a/*γ*-Fe**_**2**_**O**_**3**_ and **1b/*γ*- Fe**_**2**_**O**_**3**_), the ratio was 15.9 and 30.9, respectively, corresponding to a much stronger *T*_2_ effect compared to *T*_1_. In general, MR contrast agents that increase signal by decreasing *T*_1_ are referred to as positive (mostly paramagnetic materials), whereas if their action leads to a decrease in signal by decreasing *T*_2_, they are referred to as negative (mostly superparamagnetic materials)^37^. Therefore, the probes used in our study can be categorized as “negative” contrast agents^38^. However, it should be noted that the very strong influence of these contrast agents on *T*_2_ relaxation times led to difficulties in obtaining the signal needed for accurate quantification. The main obstacle was the minimum echo time (0.04) that could be set on the relaxometer used. The average *T*_*2*_ relaxation times of probes **1a/*γ*-Fe**_**2**_**O**_**3**_ and **1b/*γ*-Fe**_**2**_**O**_**3**_ at 10–100% concentrations were in the range of 0.3 ± 0.001–0.05 ± 0.006 ms and 0.3 ± 0.001–0.05 ± 0.001 ms, respectively, so at the limit of measurability. Another important indicator of the effectiveness of contrast agents are *r*_*1*_ and *r*_*2*_ relaxivities. These values are the main physicochemical parameters that are considered for the development of an efficient MRI contrast agent and are dependent on the size, saturation magnetization, magnetic fields and chemical structure of the molecule as well as on the accessibility of water molecules to the magnetic center. Very high *r*_2_/*r*_1_ ratios are characteristic for SPION-type superparamagnetic colloids and typically range from 6 to 15 (10-40 MHz), making lower imaging fields more suitable for exploiting the *T*_1_ effect^39^. The *r*_2_/*r*_1_ values of both colloidal probes used in our study were slightly higher than those of structurally similar Resovist®, a clinically approved iron oxide-based contrast agent for MRI (see Table 3). This demonstrates their high efficiency, which allows them to be used at lower concentrations, thus reducing their potential adverse effects in vivo.

**Table 3:** ^1^H and ^31^P relaxation properties of polymer/iron complexes. The standard deviations of all measured variables were ≤ 5%.

Sample code	^1^H *T*_1_ [ms]	^1^H *T*_2_ [ms]	^1^H *r*_1_ [s^−1^ mM^−1^]	^1^H *r*_2_ [s^−1^ mM^−1^]	*r*_2/_*r*_1_	^31^P *T*_1_ [ms]	^31^P *T*_2_ [ms]
^i^Resovist®	n.d.^ii^	n.d	12.3	188.0	15.3	n.d	n.d
*γ*-Fe_2_O_3_	41.9	1.2	7.4	259.2	35.0	n.d	n.d
1a/Fe^3+^	16.0	9.7	2178.9	3599.7	1.7	< 200	11.7
1b/Fe^3+^	10.3	6.2	1209.0	2003.0	1.7	< 200	3.1
1a/*γ*-Fe_2_O_3_	0.8	0.1	181.8	2897.4	15.9	n.d	n.d
1b/*γ*-Fe_2_O_3_	1.5	0.1	102.9	3182.4	30.9	n.d	n.d

^i^The relaxivity values were taken from the literature. ^34^.

^ii^Not detectable.

In ^31^P MR relaxometry study, we were able to measure *T*_2_ relaxation times only for **1a/Fe**^**3+**^ and **1b/Fe**^**3+**^ complexes, while *T*_1_ measurements could not be performed due to the inability to achieve a sufficiently short Repetition Time (TR) for capturing the relaxation curve using the saturation recovery method. This resulted in saturation of MR signal even at the shortest possible TR. Based on these results, we can only conclude that the ^31^P *T*_1_ is significantly shorter than 200 ms. The same was true for phantoms containing polymer/*γ*-Fe_2_O_3_ complexes, whose relaxation times were also too short to be reliably assessed (see Table 3). To summarize the results from ^31^P MR relaxometry, probes based on phospho-polymers coordinated with iron have significantly shorter relaxation times compared to phospho-polymers alone, whose *T*_1_ is typically in the range of 1000–2100 ms and *T*_2_ in the range of 30–200 ms^17,18^.

**Figure 3:**
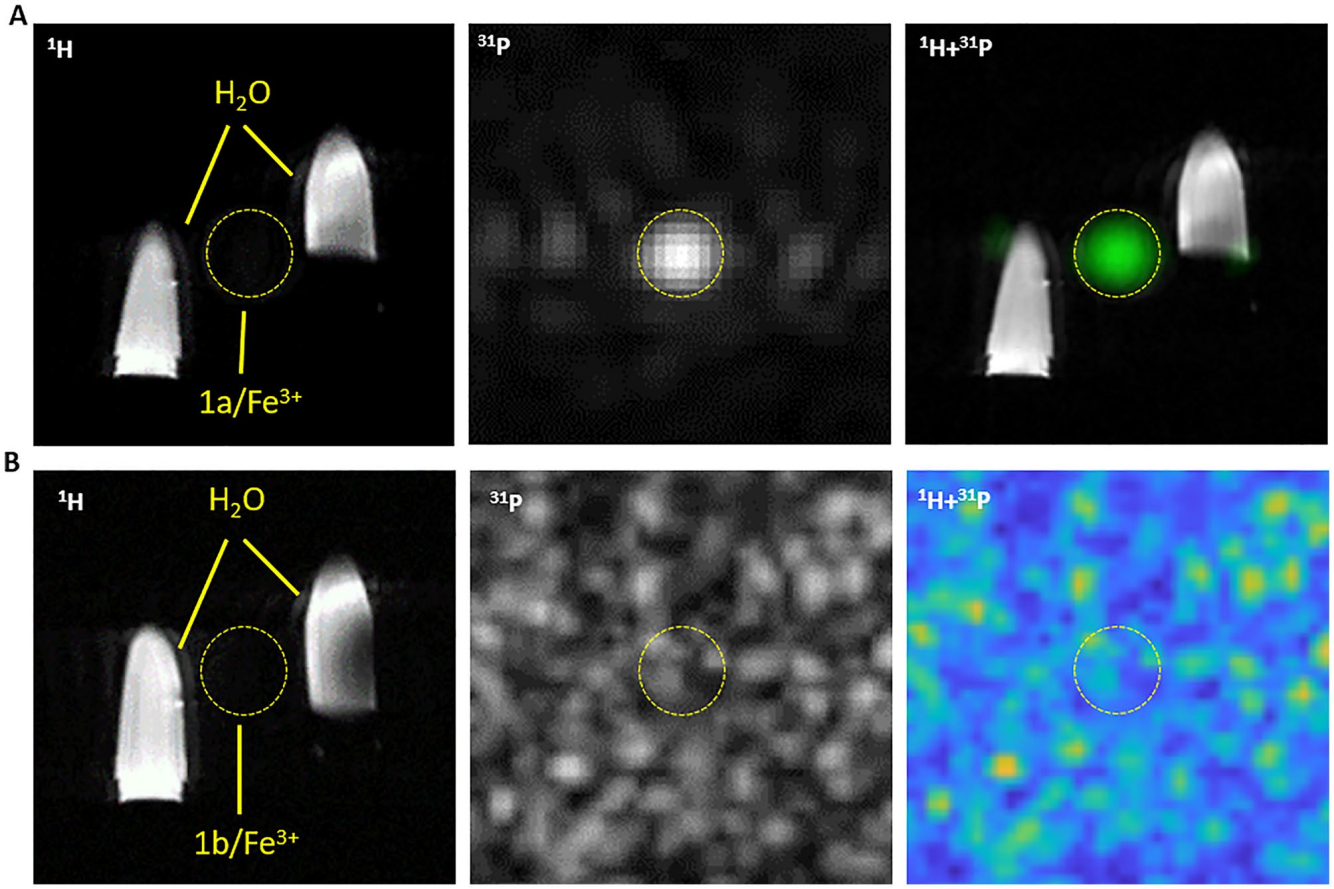
^1^H MRI (on the left), ^31^P MR CSI (in the middle) and overlapped ^1^H MRI/^31^P MR CSI measurements (on the right) of vials with water on the sides (controls) and a vial with a solution of (**A**) **1a/Fe**^**3+**^ and (**B**) **1b/Fe**^**3+**^ complexes between them.

**Figure 4:**
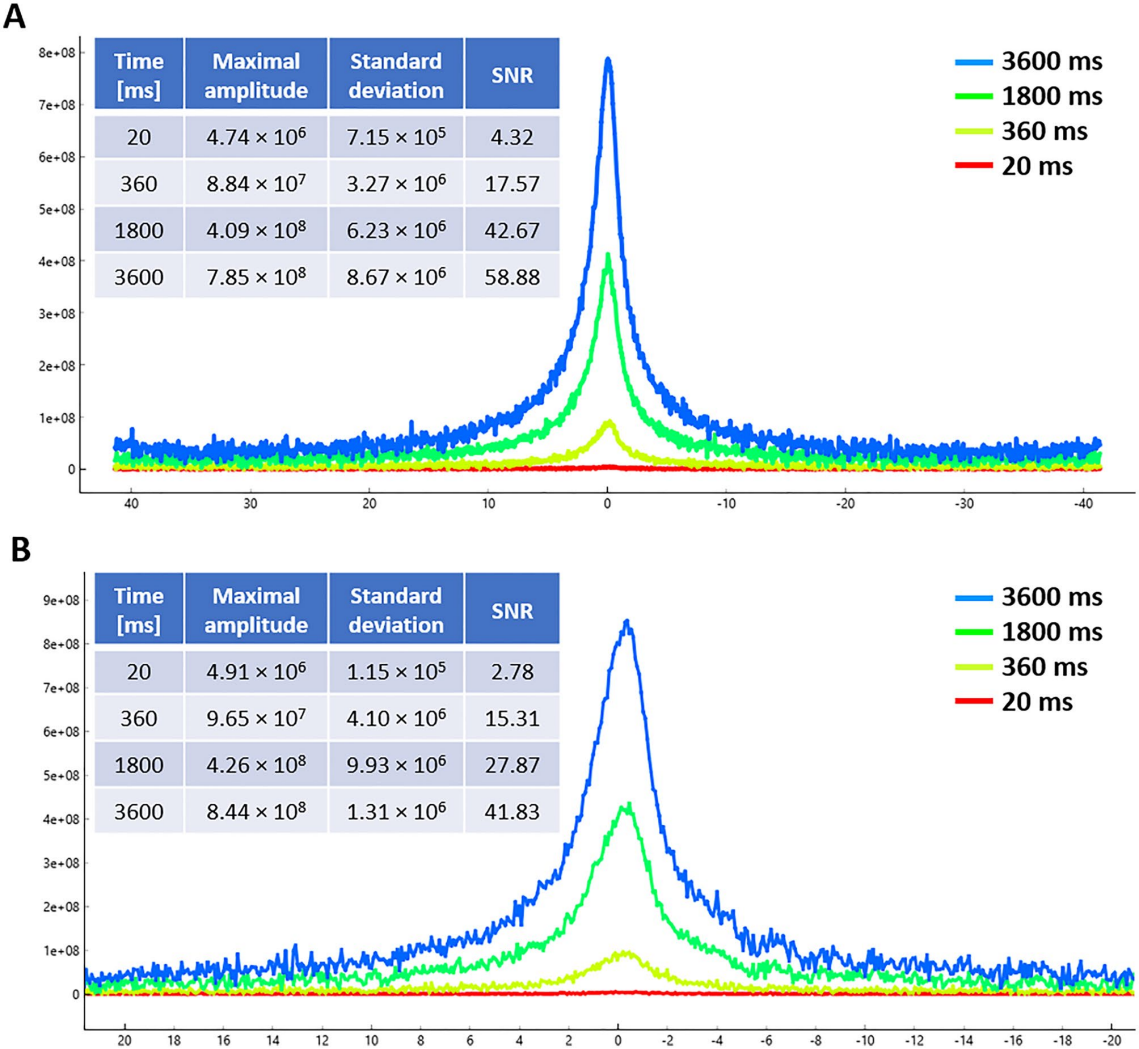
^31^P MRS measurements of aqueous solutions of (**A**) **1a/Fe**^**3+**^ and (**B**) **1b/Fe**^**3+**^ complexes at different scan lengths. The inserted tables show the numerical values of the measured data.

The conclusions resulting from the relaxometric measurement were also supported by the ^1^H MR imaging data, where a complete signal loss was observed in phantoms containing aqueous solutions of both phospho-polymers coordinated with small paramagnetic Fe^3+^ ions (**1a/Fe**^**3+**^and **1b/Fe**^**3+**^) on *T*_2_-weighted images (Figure 3). In ^1^H MR spectroscopy, a more sensitive method, signal detection was achieved in phantoms containing both polymeric **1a/Fe**^**3+**^ and **1b/Fe**^**3+**^ complexes, although a lower signal-to-noise ratio and a broader peak were observed for the **1b/Fe**^**3+**^ probe with higher content of iron (Figure 4). In the case of phantoms containing polymer/*γ*-Fe_2_O_3_ nanoparticle complexes (**1a/*γ*-Fe**_**2**_**O**_**3**_ and **1b/*γ*- Fe**_**2**_**O**_**3**_), the relaxation times were so short that both imaging and spectroscopic measurements were not feasible for technical reasons, as insufficient signal was obtained for proper measurement setup. This is consistent with the very short *T*_1_ and, particularly, the ultrashort *T*_2_ relaxation times (~ 0.1 ms) obtained for those complexes by ^1^H MR relaxometry.

Although a complete loss of proton signal was observed for both polymeric complexes (**1a/Fe**^**3+**^ and **1b/Fe**^**3+**^) in ^1^H MR experiments, the phosphorus signal in ^31^P MR measurement still remained detectable. However, the complex of semitelechelic polymer with one iron atom per chain (**1a/Fe**^**3+**^) provided a high signal well detectable by both spectroscopy and imaging, while the complex of multivalent polymer with several iron atoms along the chain (**1b/Fe**^**3+**^) was detectable only by the more sensitive spectroscopic technique (Figure 4). Expectably, the iron concentration in both *γ*-Fe_2_O_3_ nanoparticle complexes was so high that no signal could be obtained from either MRS or MRI measurements. It suggests that the most suitable structure and composition of all studied probes has a water-soluble complex of semitelechelic polymer (**1a/Fe**^**3+**^), which has a sufficient concentration of phosphorus and iron to enable efficient dual ^1^H and ^31^P MR imaging. At the same time, the concentration of iron in the probe is so low that the toxic effect of the complex is very unlikely. Moreover, the easy copolymerizability of pMPC with functionalized methacrylate/mathacrylamide-based monomers (e.g. 3-(3-methacrylamidopropanoyl)thiazolidine-2-thione, *N*-(3-aminopropyl)methacrylamide, *N*-(3,4-dihydroxyphenethyl)methacrylamide, etc.) allows the post-polymerization introduction of various bioactive compounds (e.g. cancerostatic drugs, immunostimulatory molecules, nucleic acids, etc.) into its structure, extending the diagnostic potential of this probe to therapeutic applications. In addition to the diagnostic use of the probe in pharmacokinetic studies or in the monitoring of transplanted cells, it can be used, for example, in the treatment of tumor diseases after incorporation of a suitable low-molecular-weight cancerostatics.

## Conclusions

In conclusion, our study focused on the synthesis and characterization of phospho-polymer/iron complexes as sensitive probes/contrast agents for dual ^1^H/^31^P MR imaging and spectroscopy. Specifically, we used RAFT polymerization technique to produce well-defined biocompatible (co)polymers based on 2-methacryloyloxyethyl phosphorylcholine (MPC) coordinated with small paramagnetic Fe^3+^ ions or superparamagnetic maghemite (*γ*-Fe_2_O_3_) nanoparticles via deferoxamine group linked to the ends or along the polymer chains. While water-soluble polymer probes with Fe^3+^ ions were used in this comparative study as a safer alternative to commonly used compounds containing Gd^3+^ ions, polymer-colloidal probes with iron in the form of maghemite served as a more effective and stable version of clinically approved nanoparticlulate iron oxide compounds. We have clearly demonstrated that the presence of iron—whether in the form of a soluble ion or a colloidal oxide—significantly affected the contrast of the imaged area in ^1^H MRI experiments. Particularly in the case of maghemite-based probes, the *r*_2_/*r*_1_ values were slightly higher than those of the structurally similar clinically approved *T*_2_ contrast agent Resovist®, indicating their high efficacy, which allows them to be used at lower concentrations and thus reduce their potential side effects. Coordinated iron also had a significant impact on the relaxation times of phosphorus, while we showed that the structure of the phospho-polymer and the form of the iron play an important role. We showed that the polymer with multiple Fe^3+^ ions along the chain as well as the polymers coordinated to maghemite nanoparticles had excessively high iron concentration, leading to quenching of both ^1^H and ^31^P MR signals. Conversely, the most suitable composition exhibited a polymer with only one Fe^3+^ ion per chain, which was reliably detected by both ^1^H and ^31^P MR imaging and spectroscopy. We believe that this dual ^1^H/^31^P MR probe could be highly valuable in various clinical and research applications where it could be used to monitor probe biodistribution while visualizing body anatomy. It could play a key role, for example, in the optimization of transplant procedures or in the monitoring of accumulation in solid tumors.

## Data Availability

The datasets used and/or analysed during the current study available from the corresponding author on reasonable request.
